# Mental health after compounding natural hazard exposure in New South Wales from 2019 to 2023: a cross-sectional study in Australian youth

**DOI:** 10.1038/s41598-026-51536-5

**Published:** 2026-05-06

**Authors:** Sarah E. Williams, Suzane M. Cosh, Amy D. Lykins, Chloe Hurrell, Warren Bartik, Phillip J. Tully

**Affiliations:** 1 School of Psychology, Adelaide University, Adelaide, Australia; 2 School of Psychology, University of New England, Armidale, Australia; 3 School of Psychology, Deakin University, Waurn Ponds, Australia; 4 School of Medicine, Adelaide University, Adelaide, Australia

**Keywords:** Climate change, Anxiety, Climate change anxiety, Mental health, Adjustment disorder, Natural disasters, Disaster medicine, Environmental sciences, Psychology, Psychology, Risk factors

## Abstract

Direct exposure to fires and floods adversely impacts mental health, although less is known about youth wellbeing after compounding natural hazards in short time frames. A youth sample aged 16–25 years completed self-report measures of adjustment disorder, alcohol/substance misuse and dependence, depression, anxiety and stress symptoms, and climate change anxiety after drought, bushfire and flood events in New South Wales. From 877 respondents, direct exposure to compounding (i.e., ≥ 2) natural hazards occurred in 182 persons and single hazard in 267. Compounding hazards increased odds for alcohol/substance misuse and dependence (adjusted odds ratio [OR] 2.19, 95% confidence interval [CI] 1.39–3.45) and anxiety (adjusted OR 1.55, 95% CI 1.04–2.30) by comparison to single hazard exposure. There was no interaction between compound hazard and life stressors (quantified by the Adjustment Disorder New Module-8). The association between compounding hazard exposure with mental health was mediated by climate change anxiety; full mediation for adjustment disorder, depression, anxiety, and stress, and partial mediation for alcohol/substance misuse and dependence. Compounding natural hazard exposure may have specific rather than global mental health impacts and is mediated by climate change anxiety. Youth may require specific support to recover from compounding hazards to promote wellbeing and manage climate change anxiety.

**Supplementary Information:**

The online version contains supplementary material available at 10.1038/s41598-026-51536-5.

## Introduction

Anthropogenic climate change is coinciding with an increasing frequency and intensity of natural hazards^1,2^,including floods, fires, and storms^3^. Apart from direct impact to livestock, infrastructure and socio-economic activities^4^,natural hazards directly impact human health encompassing mortality, communicable disease, ambient particulate matter, displacement and trauma among others^2,3^. Accordingly, many international organisations and disaster stakeholders agree that climate change poses a leading threat to human health and wellbeing^3,5–7^. A bushfire impacting 150,000 people results in annualised economic burden of AUD $337 million attributable to mental health alone^8^,underscoring the importance of identifying risk factors for poorer mental health after natural hazards^9^..

Natural hazards deleteriously impact mental health, increasing the risk for depression and anxiety^10^, alcohol/substance misuse and dependence^11^, death by suicide^9^and ongoing trauma symptoms^12^. The average length of trauma recovery after natural hazards is 12 to 24 months^13^,though trauma symptoms from exposure during childhood may persist to adulthood^14^. Distress following natural hazards may depend on the complex interplay between pre-, peri- and post-exposure factors^15,16^. For example, traumatic dissociation and post-traumatic stress disorder (PTSD) symptoms are higher among people who themselves or have family physically injured^17^. Higher anxiety and depression symptoms are associated with displacement due to property damage^18^ and disruption to basic infrastructure^19^and civic institutions such as schools^20^. The onset of distress symptoms following exposure to natural hazard may parallel the onset of the hazard itself, being typically rapid onset after wildfires/bushfires by comparison to insidious onset drought^21^. Collective findings indicate that natural hazards impinge on mental health, though less is known regarding the impact of compounding hazard exposures (i.e., two or more natural hazards occurring simultaneously or in close succession in a geographical region) and especially among youth populations^20,22^. Given the predicted increased severity and frequency of natural hazards^2,3^,further research among youth is required to identify adverse mental health impacts and inform planning and preparedness interventions and resourcing^20^.

Children and young people are consistently identified as vulnerable to both the long-term adverse effects of climate change^23^and trauma^24^. Brain maturation throughout childhood and young adulthood occurs in cognitive processes of reasoning, decision making, self-control, risk taking and emotion regulation^25–27^. Ongoing brain maturation in cognitive processes and connectivity between brain regions may leave adolescents particularly vulnerable to trauma^24,28^,with young people having developed fewer effective coping skills to shield themselves from negative stimuli than adults^29^. Thapa and colleagues^30^ recently argued that exposure to extreme weather events should be classified as adverse childhood experiences in accordance with recognised stressors such as physical and sexual abuse. Along these lines, recovery from compounding natural hazards, in combination with other stressors, may pose especially challenging for youth. The Lancet Commission on adolescent health and wellbeing^5^ identified rising rates in mental disorders and compounding megatrends, such as climate change and environmental degradation as key threats to youth wellbeing. Repeat natural hazards have an accumulative effect on distress symptoms in children^22^and may increase threat perception to natural hazards in adolescents^31^. Such findings parallel those among adults, where compounding events are associated with poorer mental health^32^. However, compounding hazard literature among youth is more sparse, reported mainly among children and adolescents^20,22,32^by comparison to the young adult literature^33–37^. Moreover, it is unclear whether compound natural hazards and other life stressors interact^38,39^ to portend poorer mental health in youth during periods of brain maturation. Therefore, the current paper compares the mental health of youth exposed to compounding natural hazards to their counterparts exposed to a single natural hazard.

## Results

The sample (*N* = 449) mean age was 21.0 ± 2.58 years and comprised 351 females (78.2%) with 59.5% of participants living in rural locations. Of the nine ethnicities participants identified with, the most common were European Australian or New Zealander (73.9%), Aboriginal and Torres Strait Islander peoples (11.4%), or Asian (9.1%). The group of participants reporting direct exposure to one natural hazard type in the past three years (*n* = 267, 59.5%) was comprised by bushfire (*n* = 52, 19.5%), drought (*n* = 40, 15%), and flood (*n* = 175, 65.5%). The group of participants reporting direct exposure to two natural hazard types in the past three years (*n* = 141, 31.4%) were comprised by bushfire (*n* = 97, 68.8%), drought (*n* = 58, 41.1%), and floods (*n* = 126, 89.4%). A total of 41 persons (9.1%) reported direct exposure to all three natural hazard types in the past three years.

### Hazard exposure and risk for distress

**Fig. 1:**
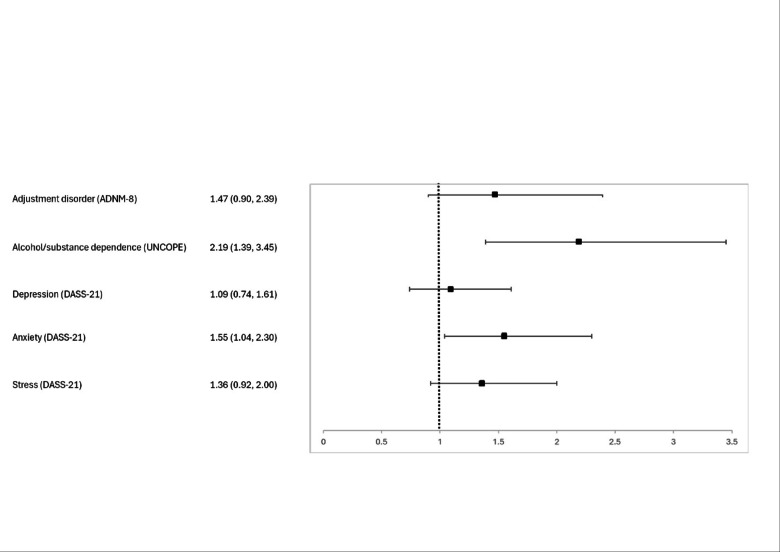
Odds for probable distress attributable to compound hazard exposure. Forest plot showing the adjusted odds ratio (square symbol) and 95% confidence interval (error bar width) for distress attributable to compound hazard exposure versus single hazard exposure (reference group). Vertical line represents no risk and odds ratios to the right of the vertical line denote increased risk. Models are adjusted for age, male gender, living rural location, and family affluence. ADNM-8, Adjustment Disorder New Module-8; CI, confidence interval; DASS-21, Depression Anxiety Stress Scales-21; OR, odds ratio; SE, standard error; UNCOPE, Unintended use Neglect Cut down Objections Preoccupation and Emotional distress.

**Fig. 2:**
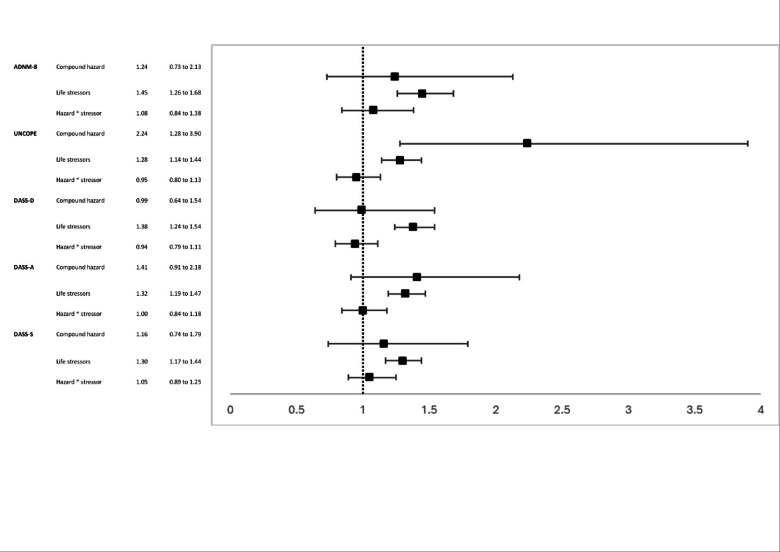
Odds for probable distress attributable to compound hazard exposure, life stressors, and compound hazard by life stressor interaction term. Forest plot showing the adjusted odds ratio (square symbol) and 95% confidence interval (error bar width) for distress attributable to compound hazard exposure versus single hazard exposure (reference group), life stressors (continuous variable), and a compound hazard by life stressor (centred) interaction term. Vertical line represents no risk and odds ratios to the right of the vertical line denote increased risk. Models are adjusted for age, male gender, living rural location, and family affluence. ADNM-8, Adjustment Disorder New Module-8; CI, confidence interval; DASS-A, Depression Anxiety Stress Scales-21 Anxiety subscale; DASS-D, Depression Anxiety Stress Scales-21 Depression subscale; DASS-S, Depression Anxiety Stress Scales-21 Stress subscale; OR, odds ratio; SE, standard error; UNCOPE, Unintended use Neglect Cut down Objections Preoccupation and Emotional distress.

Youth scoring above relevant clinical thresholds were common for adjustment disorder (348/449, 77.5%), alcohol/substance misuse (105/449, 23.4%), mild depression (244/449, 54.3%), mild anxiety (260/449, 57.9%) and mild stress (232/449, 51.7%). Compound hazard exposure, versus single hazard exposure, was associated with a two-fold increase in odds for probable alcohol/substance misuse and dependence and a 55% increase in odds for mild anxiety in adjusted analyses (Fig. 1). Given the association between compound hazard with alcohol/substance misuse and dependence, and not reaching significance for adjustment disorder, ancillary analysis explored compound hazard in relation to probable dual diagnosis (i.e. scoring above threshold for adjustment disorder and alcohol/substance misuse and dependence). Using multinomial regression, cumulative hazard exposure was associated with odds for dual diagnosis (OR 2.73, 95% CI 1.44 to 5.18) but not for a single diagnosis (OR 1.35, 95% CI 0.79 to 2.32). Analyses entering cumulative life stressors on the ADNM-8 and an interaction term for compound hazard * life stressors indicated that the cumulative number of life stressors was consistently associated with 28–45% higher odds for clinically relevant symptom thresholds of adjustment disorder, alcohol/substance misuse and dependence, depression, anxiety and stress (Fig. 2). The confidence intervals attributable to compound hazards generally overlapped those attributable to life stressors. Moreover, none of the compound hazard * life stressors interaction effects were significantly associated with the psychological outcomes. Taken together, the findings suggest that compound hazard exposure has a rather specific pattern of association with mental health whereas life stressors have a more universal impact.

### Association between hazard exposure and distress

A comparison between hazard groups indicated that youth exposed to compounding hazards reported poorer mental health than youth exposed to a single hazard in measures of alcohol/substance misuse and dependence and anxiety and all effect sizes were small or negligible (Table 1). Significant differences between hazard exposure were also observed for climate change anxiety and its subscales, though all effect sizes were small. Linear regression models provided similar findings, indicating a small portion of variance in mental health was explained by exposure to compounding hazards (Table 2). Parallel mediation analyses showed that natural hazard exposure’s effect on adjustment disorder symptoms and DASS-21 scores was fully mediated by climate change anxiety, with non-significant direct effects observed by contrast to significant indirect effects (Table 3). However, for alcohol/substance misuse and dependence, there was evidence for partial mediation, with multiple hazard type exposure still having a direct effect (*p* =.01) in addition to the indirect effect (bootstrapped β = 0.06, 95% CI 0.01 to 0.15).

**Table 1:** Between hazard group comparison on mean distress scores with ANOVA.

	Single hazard exposure type *N* = 267	Multiple hazard exposure *N* = 182	F	df_1_, df_2_	η_*p*_^2^	*P*
Alcohol/substance misuse and dependence (UNCOPE)	1.67 ± 1.88	2.19 ± 2.09	8.00	1, 443	0.02	0.005
Depression (DASS-21)	8.10 ± 5.97	8.45 ± 5.93	0.68	1, 443	0.00	0.41
Anxiety (DASS-21)	7.10 ± 5.33	8.05 ± 5.17	4.50	1, 443	0.01	0.034
Stress (DASS-21)	9.49 ± 5.25	10.42 ± 5.01	2.74	1, 443	0.01	0.098
Climate change anxiety (CCAS)	22.78 ± 9.24	24.92 ± 10.51	10.40	1, 442	0.02	0.001
Climate change cognitive emotional impairment (CCAS)	14.62 ± 5.86	15.97 ± 6.68	10.12	1, 443	0.02	0.002
Climate change functional impairment (CCAS)	8.16 ± 3.79	8.99 ± 4.35	9.39	1, 442	0.02	0.002
Experience of climate change (CCAS)	7.87 ± 3.15	8.96 ± 3.48	13.73	1, 443	0.03	< 0.001
Behavioural engagement (CCAS)	22.82 ± 4.04	22.66 ± 4.26	0.15	1, 442	0.00	0.70
Adjustment disorder (ADNM-8)	22.57 ± 5.60	23.59 ± 5.19	3.57	1, 445	0.01	0.059

Factorial ANOVA adjusted for age, gender, living rural vs. metropolitan, and family affluence; ADNM-8, Adjustment Disorder New Module-8; CCAS, Climate Change Anxiety Scale; DASS-21, Depression Anxiety and Stress Scales-21; UNCOPE, Unintended use Neglect Cut down Objections Preoccupation and Emotional distress.

**Table 2:** Multiple hazard exposure linear association with measures of distress and climate change anxiety.

Outcome	_unst_B	SE B	_st_B	_s_ *r*	*P*	Lower 95% CI for _st_B	Upper 95% CI for _st_B	*R* ^2^
Adjustment disorder (ADNM-8)	1.00	0.53	0.09	0.09	0.059	− 0.04	2.05	0.04
Substance misuse and dependence (UNCOPE)	0.57	0.19	0.14	0.14	0.004	0.19	0.95	0.01
Depression (DASS-21)	0.47	0.57	0.04	0.04	0.41	− 0.65	1.60	0.04
Anxiety (DASS-21)	1.08	0.51	0.10	0.10	0.034	0.08	2.08	0.03
Stress (DASS-21)	0.83	0.50	0.08	0.08	0.098	− 0.16	1.81	0.03
Climate change anxiety (CCAS)	3.01	0.93	0.15	0.15	0.001	1.18	4.84	0.07
Climate change cognitive emotional impairment (CCAS)	1.89	0.59	0.15	0.15	0.002	0.72	3.05	0.07
Climate change functional impairment (CCAS)	1.17	0.38	0.14	0.14	0.002	0.42	1.93	0.07
Experience of climate change (CCAS)	1.19	0.32	0.18	0.17	< 0.001	0.56	1.82	0.05
Behavioural engagement (CCAS)	− 0.16	0.40	− 0.02	− 0.02	0.70	− 0.95	0.63	0.03

Multivariate regression adjusted for age, gender, living rural location, family affluence; ADNM-8, Adjustment Disorder New Module-8; CCAS, Climate Change Anxiety Scale; CI, confidence interval; DASS-21, Depression Anxiety and Stress Scales-21; UNCOPE, Unintended use Neglect Cut down Objections Preoccupation and Emotional distress.

**Table 3:** Relationship between compound hazards and mental health through climate change anxiety mediator.

Predictor (X)	Mediator (M)	Outcome (Y)	Total effect β (SE) *p*	Direct Effect β (SE) *p*	Hazard-Mediatorβ	Mediator-Outcomeβ	Indirect effect^2^ β (SE) 95% CI
Compound hazard exposure	Climate change anxiety (CCAS)	Adjustment disorder (ADNM-8)^1^	1.02 (0.53) 0.05	0.70 (0.52) 0.17	2.23 (0.94) 0.02*	0.14 (0.03) <0.001**	0.32 (0.14) 0.07 to 0.64
		Depression (DASS-21)	0.38 (0.57) 0.51	− 0.09 (0.54) 0.87	2.14 (0.94) 0.02*	0.22 (0.03) <0.001**	0.47 (0.23) 0.05 to 0.94
		Anxiety (DASS-21)	0.97 (0.51) 0.06	0.49 (0.46) 0.29	2.14 (0.94) 0.02*	0.23 (0.02) <0.001**	0.48 (0.23) 0.06 to 0.96
		Stress (DASS-21)	0.91 (0.50) 0.07	0.52 (0.47) 0.27	2.14 (0.94) 0.02*	0.18 (0.02) <0.001**	0.39 (0.18) 0.05 to 0.77
		Substance misuse and dependence (UNCOPE)	0.53 (0.19) 0.01*	0.47 (0.19) 0.01*	2.14 (0.94) 0.02*	0.03 (0.01) 0.01*	0.06 (0.04) 0.01 to 0.15

1. *N* = 440 for ADNM-8 analyses, otherwise *N* = 448.

2. Indirect effects were computed using bootstrapping of 10,000 resamples.

ADNM-8, Adjustment Disorder New Module-8; CCAS; Climate Change Anxiety Scale; CI, confidence interval; DASS-21, Depression Anxiety and Stress Scales-21; SE, standard error; UNCOPE, Unintended use Neglect Cut down Objections Preoccupation and Emotional distress; * *p* <.05, ** *p*<.01.

## Discussion

This study demonstrated that exposure to compounding natural disasters was associated with elevated symptoms of alcohol and substance misuse and dependence, and anxiety among youth. In a sample already exposed to natural hazards, there was no evidence for an interaction effect between compound hazard and life stressors. Life stressors themselves were associated with 28–45% increased odds for adjustment disorder, substance misuse, depression, anxiety and stress. While life stressors were associated with a broad range of mental health outcomes—conferring a 28–45% increased odds across multiple domains—compound hazard exposure showed a more circumscribed pattern. Further, a series of mediation analyses also identified that the association between compound hazards and mental health was partially (in the case of alcohol/substance misuse and dependence) or fully (in the case of adjustment disorder, depression, anxiety, and stress symptoms) mediated by climate change anxiety.

These findings align with prior research linking cumulative disaster exposure with heightened anxiety and posttraumatic stress symptoms^35^. However, youth exposed to compounding disasters were not uniformly more likely to meet criteria for probable adjustment disorder alone; rather, they were at two-fold elevated risk for dual diagnosis presentations involving both adjustment disorder and alcohol or substance misuse or dependence. This pattern may reflect sustained hypervigilance, heightened sensitivity to environmental threat, and maladaptive coping following repeated hazard exposure. Longitudinal evidence indicates that recurrent disaster exposure is more likely to produce increased senstitisation to hazards^33^than habituation^34^, potentially increasing vulnerability to maladaptive coping responses. Although trauma research in youth consistently identifies resilience, recovery, and chronic symptom trajectories^40^, the mechanisms underpinning resilience following compounding natural hazards exposure remain poorly understood. Consistent with prior studies^41–43^,unhealthy coping strategies such as alcohol and substance use were evident among disaster-exposed youth. Similar patterns have been observed in adult disaster-affected populations^44–46^. However, it is notable that the accumulation of non-disaster life stressors independently increased the likelihood of distress without interacting with compound hazard exposure. This absence of interaction suggests that youth may be particularly vulnerable to poly- and complex trauma and substance misuse irrespective of disaster exposure^47^, highlighting the additive rather than synergistic nature of these stress pathways.

A novel contribution of this study is the identification of climate change anxiety as a mediating pathway linking compound hazard exposure with specific mental health outcomes. The small associations between compound natural hazard exposure and symptoms of depression, anxiety and stress were fully mediated by climate change anxiety. The small magnitude associations with alcohol and substance misuse were partially mediated, indicating that compound exposure, along with climate change anxiety, were associated with alcohol and substance misuse symptoms. Our alcohol/substance misuse findings therefore support at least one recently theorised pathway^48^through which climate change is associated with substance abuse due to climate change anxiety or worry about the unchecked consequences of climate change. Heightened awareness of climate change risk and environmental vulnerability is known to follow direct and indirect exposure to natural disasters^7^, and emerging evidence links climate change awareness^49^ and climate anxiety^10^with depression, anxiety, and stress symptoms. The present findings extend this literature by demonstrating that repeated hazard exposure may amplify anticipatory threat, hypervigilance, and perceived vulnerability through climate anxiety, thereby contributing to difficulties coping with uncertainty and future risk^50^. Nonetheless, as a cross-sectional study only, it is unclear the extent to which mental health concerns and climate change anxiety were pre-existing, even at subthreshold levels, prior to the various natural hazards encountered by respondents. Thus, a tri-directional association between natural hazards, mental health, and climate change anxiety may occur. This interaction could potentially be explored through structural equation modelling and longitudinal designs.

These findings have important implications for the resourcing and delivery of youth mental health services following natural disasters. In the immediate aftermath of disasters, mental health care is often difficult to implement and triage effectively^51^. In Australia, post-disaster mental health supports are typically delivered through disaster-relief agencies and non-government organisations and are frequently short-term in nature^52,53^. As climate change continues to intensify and compounding hazard exposure becomes increasingly likely, additional and sustained resourcing will be required to support youth recovery and preparedness. The present results suggest that alcohol and substance misuse and dependence and anxiety including climate change anxiety represent specific mental health needs following compounding natural hazards. These targeted impacts may not be adequately detected using brief, global measures of psychological distress such as the Kessler Psychological Distress Scale-6^54^, the Mental Health Inventory-5^32^,or the Short Form-12 Health Survey^36^. While such instruments may be useful for large-scale post-disaster screening, they lack the specificity required to inform substance-focused and trauma-informed interventions^43^. Incorporating measures that assess adjustment, substance use, and climate-related anxiety may improve identification of youth with specific clinical treatment needs.

Strengths of this study include a large sample of youth exposed to one or more natural disasters within a relatively short timeframe across a single Australian state. Nonetheless, several limitations warrant consideration. Flood events were temporally more recent than drought and bushfire exposure and may therefore have been more salient to participants. Prior research suggests that disaster exposure within the previous two to three years represents a particularly high-risk period for mental distress in school-aged children^20^. Recall bias may also have influenced reporting, particularly for insidious hazards such as drought compared with more acute events like bushfires. The analytical approach excluding persons without natural hazard exposure may also lead to collider restriction biases, a form of selection bias^55^. Although recruitment strategies aimed to capture geographic and cultural diversity, there was an overrepresentation of female participants, which future research should seek to address^56^. The study managed to recruit a sample with 11.4% of respondents identifying as Aboriginal and Torres Strait Islander Peoples. Though this is higher than the 3.4% NSW consensus estimate, the oversampling rate possibly reflects the sampling from rural areas, with up to 67% of First Nations peoples residing in NSW outside of greater Sydney region^57^. Moreover, the second largest First Nations Peoples demographic residing in NSW are aged 15–24 (18.5%)^57^. Otherwise, the sample predominantly comprised youth of European ancestry residing within a single Australian state, limiting generalisability to other cultural and geographic contexts. In addition, disaster exposure was restricted to bushfire, drought, and flood, and findings may not extend to other hazard types such as cyclones, hurricanes, or earthquakes. The cross-sectional design precludes causal inference, and longitudinal studies are needed to examine temporal pathways and cumulative risk processes. The reliance on self-report measures without structured clinical interviews limits diagnostic precision, and important outcomes such as suicidality^37^ and sleep disturbance^44^ were not assessed. Moreover, other important covariates and moderators were not assessed, including employment status, political views, and belief in climate change.

In conclusion, exposure to compounding natural disasters was associated with elevated symptoms of alcohol and substance misuse, and anxiety among youth compared with exposure to a single hazard type. These associations were partly or fully mediated by climate change anxiety, highlighting a potential psychologically meaningful threat-vulnerability pathway linking repeated hazard exposure with mental health. Importantly, the mental health impacts of compound hazard exposure were relatively specific, in contrast to the more generalised effects associated with accumulating life stressors. Together, these findings underscore the value of incorporating targeted assessments of adjustment, substance use, and climate change anxiety to identify youth most in need of support following repeat natural disasters.

## Method

### Population

A sample of young people aged 16–25 years, residing in New South Wales (NSW), Australia, were recruited for an anonymous survey. This age range is used by the Australian Institute of Health and Welfare to define young people and aligns with eligibility for many government and non-government health services^58^. The study categorised respondents as major city dwelling youth versus regional centres, rural towns, and remote dwelling participants according to Modified Monash Model ([MM] MM1 vs. MM ≥ 2)^59^. Participants answered the demographic questions which formed part of the inclusion criteria (i.e. age, post code), as well as ethnicity^60^,followed by each of the survey questionnaires (described further below) in a randomised manner. Family affluence was measured using a validated six item measure (Family Affluence Scale Version III [FAS-III])^61^.

### Direct hazard exposure definition

Data was collected between December 2022 through January 2023 regarding exposure to the following disasters: severe flooding in 2020–2021; 2021–2022; 2022–2023, severe and catastrophic Black Summer fires 2019–2020, and severe drought in 2019. The recall period for hazard exposure was three years. Respondents were asked about both direct and indirect exposure to each of these natural disasters and given free-text response options to specify details for each disaster and any direct exposure, and indirect exposure. Nearly half the original sample (428/877, 48.8%) reported no direct exposure and were excluded from further analyses (sTable 1). Ineligible participants with no hazard exposure were more likely to be male, residing in a city, and of non-European cultural background. From the retained analytical sample of 449 persons, there were 267 persons (59.5%) who reported direct exposure to one natural hazard and 182 (40.5%) to two or more disaster types.

### Primary outcomes

#### Adjustment disorder

Adjustment disorder symptoms were measured using the Adjustment Disorder New Module 8 (ADMN-8)^62^.This brief self-report measure assesses the number and severity of adjustment disorder symptoms defined by the International Classification of Diseases 11th edition^63^. In Part 1, participants are asked to indicate whether they have experienced one or more of sixteen defined stressful life events, and one “other” item, that have occurred over the past 1–2 years and “burdened” the individual over the preceding 6 months (see Supplement sT2). If participants answered “yes” to at least one of these items, they were then presented with an additional 4 items assessing preoccupation and another 4 items assessing failure to adapt^38^. Example prompts about recent impact e.g., “Since the stressful situation, I do not like going to work or carrying out the necessary tasks in everyday life”. The scale uses a 4-point Likert scale: 1 = never, 2 = rarely, 3 = sometimes, 4 = often. A higher total score indicates more severe adjustment symptoms. The scale has been used in previous studies with samples comprising youth and older adults and male and female participants in both a clinical sample in UK and a non-clinical sample in Switzerland^62^. Scores ≥ 19 higher indicating a probable diagnosis of adjustment disorder^64^were utilised in analyses of binary outcomes here. The internal reliabilities (Cronbach’s alphas) of the ADMN-8 scores were satisfactory for both the Switzerland and UK samples in total scores and both the failure to adapt and preoccupation subscales^62^. Reliability in the current sample was α = 0.89.

#### Alcohol/substance abuse and dependence

The UNCOPE^65^is a 6-item screening tool for the assessment of possible alcohol/substance abuse or dependence, appropriate for use in adolescent and justice populations^66^. Items reflect the UNCOPE domains of using more than intended, neglect of responsibilities, attempts to cut down, objections by others, preoccupation with use, and use to relieve emotional discomfort. Each item is endorsed ‘yes’ or ‘no’ with dichotomous responses then summed to produce a total score ranging from 0 to 6, with higher scores indicating greater risk of substance dependence. Hoffmann et al.^65^demonstrated acceptable internal reliability (α =.85) and criterion accuracy against DSM-IV dependence. A score of 4 or more symptoms was used to denote probable substance dependence and misuse in analyses of binary outcomes here. Using this cut score, the UNCOPE has showed high specificity (detecting true negatives) of 86%−97% and high sensitivity (detecting true positives) of 84%−89% for alcohol, cocaine, and marijuana^65,67^. Internal reliability in this sample was good (α =.82).

#### Depression, anxiety, stress

The Depression Anxiety Stress Scales (DASS-21)^68^assesses psychological distress over the preceding week. The DASS-21’s three subscales (depression, anxiety, and stress) consist of 7-items each. Each item is measured on a 4-point Likert scale (0 = “never” to 3 = “almost always”) with higher scores indicating increase distress on each respective subscale^69,70^. Clinical thresholds to denote mild, moderate, severe, and extremely severe ranges for each subscale are established^71^. The DASS-21 thresholds have good sensitivity and specificity for predicting diagnoses obtained from a structured interview^70,71^. Confirmatory factor analyses demonstrate that the DASS-21 has a stable three-factor psychometric structure which can be divided into features of depression, anxiety, and general distress (stress)^72^. Cronbach’s alphas in the current sample were very good for depression (α = 0.92), and good for anxiety (α = 0.88) and stress (α = 0.88).

#### Climate change anxiety

The Climate Change Anxiety Scale (CCAS)^73^assesses anxiety and psychological symptoms related to climate change in a self-report format. A 5-point Likert scale (range: 1 = “never” to 5 = “almost always”) is used to measure responses to 22 items (e.g., “Thinking about climate change makes it difficult for me to concentrate”). A summed total score of items 1 to 13 is used to quantify climate change anxiety (CCAS total) where higher scores denote greater climate anxiety^73^. Score of 21 or higher are indicative of mild to moderate distress^74^. The first 8 items are considered a measure of cognitive-emotional impairment and items 9 to 13 quantify functional impairment. Items 14 to 16 of the CCAS measure personal experience of climate change and items 17 to 22 individual behavioural engagement. Adequate reliability has been found in previous studies using the CCAS with young adults^73^. Cronbach’s alpha in the current sample was excellent for CCAS total (α = 0.94), cognitive-emotional impairment (α = 0.91), functional impairment (α = 0.90), good for personal experience of climate change (α = 0.88), and acceptable for behavioural engagement (0.73).

## Procedure

Advertising materials were circulated by Qualtrics and a link was provided to complete an informed consent sheet and the survey. Upon clicking the survey link, participants were presented with the study details and support service information. After providing informed consent, participants answered demographic questions, followed by each of the survey questionnaires in a randomised manner. The survey had 2 randomly interspersed attention questions that participants had to answer correctly to continue. Participants received a small reward for their time (e.g., points toward gift cards or charitable donations), distributed by Qualtrics. Qualtrics were responsible for confirming eligibility and reviewed the data quality, removing straight-lining respondents, speeders (< 1/3 median completion time), persons failing any of the 2 randomly interspersed attention checks. The study was approved by the Human Research Ethics Committee of the University of New England and was performed in accordance with relevant guidelines and regulations including the Declaration of Helsinki.

### Data analysis

Analyses were conducted using IBM SPSS Statistics Version 30.0 (IBM Corp., Armonk, N.Y.). The severity of distress on different DASS thresholds was compared between groups with chi-square for linear trend (see supplement sTable 3). The odds ratio (OR) and 95% confidence interval (CI) for probable distress using established thresholds was examined with logistic regression, adjusted for age, female gender, living in rural location (defined by Modified Monash Model 3 or higher)^59^ and family affluence. In ancillary analyses, the contribution of compounding life stressors, determined by ADNM-8 events, was examined as a linear predictor and centred interaction term * compound hazard.

Mean scores on continuous measures of distress (ADNM-8, UNCOPE, DASS-21 and CCAS) were compared with factorial analysis of variance (ANOVA), adjusted for age (continuous scale), female gender (= 1, male and ‘other’ code = 0), regional/rural location (= 1) and FAS-III (continuous scale). We included participants identifying from non-cis genders by adding them to the least-represented gender in our study (i.e. males) rather than exclude these participants^75^. ANOVA data are reported as M ± SD with *η*_*p*_^*2*^. Cohen categorises *η*_*p*_^*2*^effect sizes 0.01 − 0.05 as small, 0.06 − 0.13 as medium, and ≥. 14 as large^76^. The linear association between compounding hazard exposure and distress was explored with multiple regression analyses adjusted for age, female gender, rural location and family affluence scale (linear). A series of mediation analyses (sFig 1) were run to determine if climate anxiety mediated the association between hazard exposure and outcome measures ADNM-8, UNCOPE, and DASS-21. These analyses used the PROCESS Model 4 macro developed by Hayes^77^ with 10,000 bootstrap samples.

Logistic and linear regression assumption tests for independence of errors was tested with the Durbin-Watson test. The assumption of constant variance was visually inspected with scatterplot of residual vs. fitted values. The assumption of normally distributed residuals was assessed by plotting histograms of residuals and Q-Q plots. Tests for multicollinearity included inspection of variance inflation factors and tolerance values. The contribution of influential observations was tested with Cook’s distance. In the ANOVA, the assumption of normality was displayed visually as Histograms and Q-Q plots and tested statistically with the Shapiro-Wilk test. Tests for homogeneity of variance (Homoscedasticity) included Levene’s test and visual inspection of boxplots.

## Electronic Supplementary Material

Below is the link to the electronic supplementary material.

Supplementary Material 1

## Data Availability

The Ethics approval for this research does not allow for data sharing as the datasets generated and/or analysed during the current study are not publicly available due some participants age less than 18 years old but are available from the corresponding author on reasonable request.
