# Supplementary material for: Mental health after compounding natural hazard exposure in New South Wales from 2019 to 2023: a cross-sectional study in Australian youth
Source: Sci Rep. 2026 May 6;16:20903. doi: 10.1038/s41598-026-51536-5 (PMC13338153; doi:10.1038/s41598-026-51536-5)
Supplement: Supplementary file 1 — Supplementary Material 1 [file 41598_2026_51536_MOESM1_ESM.docx]

**Supplementary files for**

**Mental health after compounding natural hazard exposure in New South Wales 2019 to 2023: a cross-sectional study in Australian youth**

**sTable 1. Descriptive data for the analytical sample by comparison to the ineligible participants**

| **Descriptive variable** | **Excluded from analysis: no hazard exposure**  **N = 426** | **Included in analysis: one more hazard exposures**  **N = 449** | **Two-sided P** |
| --- | --- | --- | --- |
| Age, M ± SD | 21.2 ± 2.6 | 21.0 ± 2.6 | .28 |
| Gender |  |  | <.001 |
| Female | 281 (66%) | 351 (78.2) |  |
| Male | 141 (33.1%) | 98 (21.8) |  |
| Other | 2 (0.5%) | 0 |  |
| Prefer not to say | 2 (0.5%) | 0 |  |
| Resides in major city^1^ | 285 (66.9%) | 182 (40.5%) | <.001 |
| Family Affluence Scale^2^ | 7.51 ± 2.2 | 7.6 ± 2.0 | .57 |
| Completed education level |  |  | .98 |
| Year 10 or less | 25 (5.9%) | 40 (8.9%) |  |
| Year 11 | 43 (10.1%) | 28 (6.2%) |  |
| Year 12 | 171 (40.1%) | 174 (38.8%) |  |
| Certificate or diploma | 55 (12.9%) | 67 (14.9%) |  |
| Trade / apprenticeship | 20 (4.7%) | 25 (5.6%) |  |
| Bachelor’s degree | 104 (24.4%) | 103 (22.9%) |  |
| Postgraduate degree | 8 (1.9%) | 12 (2.7%) |  |
| Cultural background |  |  | <.001 |
| European Australian | 285 (66.9%) | 331 (73.7%) |  |
| Aboriginal and Torres Strait Islander peoples | 34 (8.0%) | 51 (11.4%) |  |
| New Zealander | 2 (0.5%) | 1 (0.2%) |  |
| Maori | 2 (0.5%) | 2 (0.4%) |  |
| Other Oceanian | 6 (1.4%) | 2 (0.4%) |  |
| Northwest European | 4 (0.9%) | 1 (0.2%) |  |
| Southern European | 2 (0.5%) | 0 |  |
| Eastern European | 2 (0.5%) | 5 (1.1%) |  |
| Southeast Asian | 35 (8.2%) | 22 (4.9%) |  |
| Northeast Asian | 15 (3.5%) | 14 (3.1%) |  |
| Southern and Central Asian | 14 (3.3%) | 5 (1.1%) |  |
| North American | 1 (0.2%) | 1 (0.2%) |  |
| South American | 5 (1.2%) | 3 (0.7%) |  |
| North African | 5 (1.2%) | 8 (1.8%) |  |
| Sub-Saharan African | 1 (0.2%) | 0 |  |
| Prefer not to say | 13 (3.1%) | 3 (0.7%) |  |

**sTable 2. Stressor events reported in the Adjustment Disorder New Module-8^3^**

| **Severity level** | **Single hazard exposure**  **N (%)** | **Compound hazard exposure**  **N (%)** | **Two-sided P** |
| --- | --- | --- | --- |
| Divorce separation | 31 (11.6%) | 34 (18.7%) | .041 |
| Family conflicts | 165 (61.8%) | 111 (61.0%) | .92 |
| Work conflicts | 128 (47.9) | 112 (61.5%) | .005 |
| Neighbour conflict | 63 (23.6%) | 37 (20.3%) | .49 |
| Illness loved one | 167 62.5%) | 131 (72.0%) | .042 |
| Death loved one | 132 (49.4%) | 102 (56.0%) | .18 |
| Retirement adjustment | 6 (2.2%) | 4 (2.2%) | 1.00 |
| Unemployment | 100 (37.5%) | 54 (29.7%) | .11 |
| Work balance | 207 (77.5%) | 137 (75.3%) | .65 |
| Time pressure | 169 (63.3%) | 134 (73.6%) | .024 |
| Moving home | 124 (46.4%) | 91 (50.0%) | .50 |
| Financial problems | 177 (66.3%) | 133 (73.1%) | .15 |
| Own illness | 56 (21.0%) | 50 (27.5%) | .12 |
| Serious accident | 25 (9.4%) | 18 (9.9%) | .87 |
| Assault | 27 (10.1%) | 21 (11.5%) | .64 |
| Termination leisure | 20 (7.5%) | 27 (14.8%) | .018 |
| Another event | 32 (12.0%) | 21 (11.5%) | 1.00 |
| **Summary** | **M = 6.10 ± 2.86** | **M = 6.69 ± 2.71** | **.32** |

**sTable 3. Severity of depression, anxiety and stress by hazard exposure**

| **Severity level** | **Single hazard exposure**  **N = 267** | **Compound hazard exposure N = 182** | **P for trend** |
| --- | --- | --- | --- |
| Depression |  |  | 0.67 |
| Normal | 93 (34.8%) | 58 (31.9%) |  |
| Mild | 30 (11.2%) | 24 (13.2%) |  |
| Moderate | 48 (18.0%) | 37 (20.3%) |  |
| Severe | 43 (16.1%) | 20 (11.0%) |  |
| Extremely severe | 53 (19.9%) | 43 (23.6%) |  |
| Anxiety |  |  | 0.036 |
| Normal | 91 (34.1%) | 43 (23.6%) |  |
| Mild | 32 (12.0%) | 23 (12.6%) |  |
| Moderate | 30 (11.2%) | 24 (13.2%) |  |
| Severe | 28 (10.5%) | 23 (12.6%) |  |
| Extremely severe | 86 (32.2%) | 69 (37.9%) |  |
| Stress |  |  | 0.11 |
| Normal | 100 (37.5%) | 54 (29.7%) |  |
| Mild | 37 (13.9%) | 26 (14.3%) |  |
| Moderate | 43 (16.1%) | 37 (20.3%) |  |
| Severe | 62 (23.2%) | 41 (22.5%) |  |
| Extremely severe | 25 (9.4%) | 24 (13.2%) |  |

*Measured with Depression Anxiety and Stress Scales-21^4^*

**
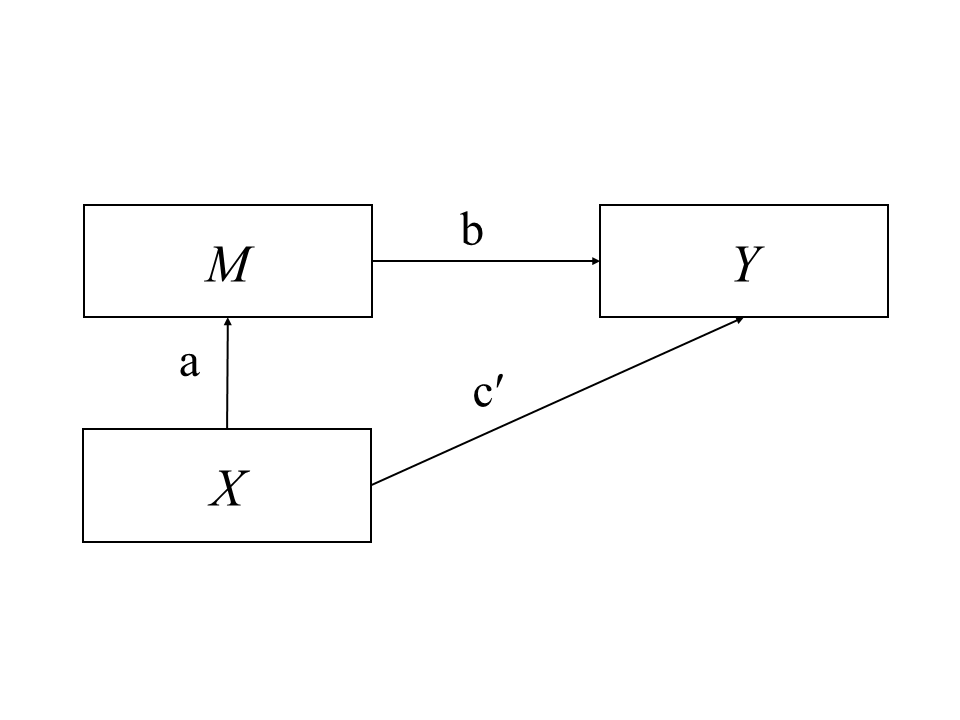
**

**sFigure 1. Mediation analysis pathway examined with PROCESS macro^5^**

The PROCESS macro Model 4 examined the predictor variable X (compound hazard exposure), mediator variable M (climate change anxiety), and outcome variables Y (symptoms of adjustment disorder, alcohol/substance misuse, anxiety) reported in Table 3. Hazard → Climate Change Anxiety pathway denoted a, Climate Change Anxiety → Outcome pathway denoted b, the indirect effect is a + b, and the direct effect of Hazard on the Outcome after accounting for the mediator is denoted c’

**Supplement references**

1 Australian Government Department of Health. Modified Monash Model. (Canberra, ACT, 2025).

2 Currie, C., Alemán Díaz, A. Y., Bosáková, L. & de Looze, M. The international Family Affluence Scale (FAS): Charting 25 years of indicator development, evidence produced, and policy impact on adolescent health inequalities. *SSM Popul Health* **25**, 101599, doi:10.1016/j.ssmph.2023.101599 (2024).

3 Kazlauskas, E., Gegieckaite, G., Eimontas, J., Zelviene, P. & Maercker, A. A Brief Measure of the International Classification of Diseases-11 Adjustment Disorder: Investigation of Psychometric Properties in an Adult Help-Seeking Sample. *Psychopathology* **51**, 10-15, doi:10.1159/000484415 (2018).

4 Lovibond, S. H. & Lovibond, P. F. *Manual for the Depression Anxiety Stress Scales (2nd Ed)* (Psychology Foundation Monograph, 1995).

5 Hayes, A. F. *Introduction to mediation, moderation, and conditional process analysis: A regression-based approach*. (Guilford Press, 2013).
